# Present and future evolution of advanced breast cancer therapy

**DOI:** 10.1186/bcr2572

**Published:** 2010-10-22

**Authors:** Ricardo H Álvarez

**Affiliations:** 1 Department of Breast Medical Oncology, The University of Texas M.D. Anderson Cancer Center, 1155 Herman P.Pressler, CPB5.3458, Houston, TX 77030-3721, USA

## Abstract

Although the introduction of novel therapies and drug combinations has improved the prognosis of metastatic breast cancer, the disease remains incurable. Increased knowledge of the biology and the molecular alterations in breast cancer has facilitated the design of targeted therapies. These agents include receptor and nonreceptor tyrosine kinase inhibitors (epidermal growth factor receptor family), intracellular signaling pathways (phosphatidylinositol-3-kinase, AKT, mammalian target of rapamycin) angiogenesis inhibitors and agents that interfere with DNA repair (poly(ADP-ribose) polymerase inhibitors). In the present review, we present the most promising studies of these new targeted therapies and novel combinations of targeted therapies with cytotoxic agents.

## Introduction

Current research in breast cancer is being guided by the discovery of multiple targets cells or tissues that have receptors for a particular hormone or drug. These targets are leading to treatments more sophisticated than conventional cytotoxic chemotherapy or hormone-based therapy. Targeting of human epidermal growth factor receptor 2 (HER2) with trastuzumab and of vascular endothelial growth factor (VEGF) with bevacizumab in combination with chemotherapy has proven to be a milestone in molecular targeted therapy for breast cancer.

As many novel targets are being discovered, multiple approaches to anticancer therapy are emerging in the literature. These approaches, referred to as targeted therapies, consist of targeting the malignant cell signal transduction machinery, including the crucial processes involved in cell invasion, cell metastasis, apoptosis, the cell cycle, and tumor-related angiogenesis. Among these therapies, a class of compounds that has shown great promise is one that targets tyrosine kinases, which are carried by small molecules or monoclonal antibodies. Intrinsic and acquired resistance to endocrine and/or cytostatic treatments, however, is still a common feature that limits the benefits for these novel therapeutic strategies. Clinical trials of endocrine or cytotoxic therapies, combined with growth factor pathway inhibitors or their downstream signaling elements, are therefore warranted. In the present review, we describe the most promising studies using these new molecular agents and their novel combinations with traditional cytotoxic agents in targeted therapies.

## Preferred treatment schemes: sequential single-agent chemotherapy or combination chemotherapy

Breast cancer is a world health problem, and in the United States this disease is the second most common cause of cancer death in women [1]. Although breast cancer is among the most chemosensitive of the solid tumors, important improvements in survival have been achieved during the past two decades with the introduction of the new agents [1]. For patients with estrogen receptor (ER)-positive metastatic breast cancer (MBC) without visceral crisis, hormone therapy has been the preferred treatment option. The optimal timing for initiation of hormone therapy or chemotherapy, however, needs to be individualized.

Several randomized phase III studies have compared single-agent chemotherapy versus combination chemotherapy, and most have reported improved response rates and time to disease progression but minimal survival benefit. A systematic review published a decade ago, which included 15 randomized trials in the pre-taxane era, concluded that multidrug combination chemotherapy was superior to single-agent chemotherapy [2]. More recently, a meta-analysis of 37 randomized trials, which included new drugs for breast cancer treatment, showed again that a combination of chemotherapeutic agents increased the response rate (odds ratio, 1.28; 95% confidence interval (CI), 1.15 to 1.42; *P* <0.00001) and improved the time to tumor progression (hazard ratio (HR), 0.78; 95% CI, 0.73 to 0.83; *P* <0.00001), with a 12% of increase in overall survival (OS) [3].

## Chemotherapy

### Standard of care: anthracyclines and taxanes

Anthracyclines and taxanes are the most active cytotoxic drugs for the treatment of breast cancer. In the adjuvant setting, the pivotal role of anthracycline-based chemotherapy bas been established in an overview of successive randomized trials by the Early Breast Cancer Trialists’ Collaborative Group [4]. Concerns have been voiced about cardiac toxicity and potential leukemogenicity with use of anthracyclines. In the metastatic setting, the incidence of cardiac dysfunction has been related to the dose and schedule of anthracyclines [5]. Cardiac toxicity with use of anthracyclines has been associated with congestive heart failure. The risk of developing congestive heart failure is also known to increase with concomitant administration of other cytotoxic drugs, such as cyclophosphamide. Doxorubicin given at 240 to 360 mg/m^2^ has reduced the incidence of congestive heart failure to around 1.6 to 2.1% [5,6]. Data from a study of long-term survivors of childhood cancer, however, indicated that no true threshold can be determined for anthracycline-related cardiotoxicity and that the symptoms of congestive heart failure become apparent years after use of the drug [7]. Several reports have shown that the incidence of cardiac toxicity is low in women who received adjuvant anthracyclines [8,9].

The advent of taxanes provided a novel option for chemotherapy, and an early single-agent randomized trial showed that results for taxanes were similar to or perhaps slightly better than those for counterpart anthracyclines in the metastatic setting [10]. Few studies have been conducted, however, comparing anthracycline-containing and taxane-containing regimens. In one such study, Jones and colleagues suggested that docetaxel plus cyclophosphamide was superior to Adriamycin plus cyclophosphamide in the adjuvant treatment of breast cancer [11]. Comparisons of docetaxel plus cyclophosphamide with Adriamycin plus cyclophosphamide represent comparisons of first-generation regimens with third-generation regimens. To date, the data are insufficient to recommend replacing anthracyclines in the adjuvant treatment of breast cancer [12].

### Newly approved chemotherapy agents: epothilones and ixabepilone

Microtubules play a crucial role in diverse cellular function including growth, motility, trafficking of vesicles, and cellular shape maintenance. The mitotic spindles – where chromosomes are attached and then separated – are composed of both α-tubulin and β-tubulin subunits, and the process of polymerization and depolymerization of the microtubule is very complex and dynamic. Two families of chemotherapeutic agents, the vinca alkaloids and taxanes, interact with microtubules. Among the taxane family, paclitaxel and docetaxel are the most widely used agents in the metastatic setting, with response rates of 32 to 68% when used as single agents [13]. Although the introduction of these agents marked a significant advance for the treatment of cancer, their clinical utility is often limited by the development of drug resistance. This resistance can be intrinsic or acquired after the tumor is exposed to certain chemotherapeutic agents. One common mechanism of tumor resistance occurs through expression of multidrug-resistance proteins (p-glycoprotein and MDR-1). These proteins build up efflux pumps, which prevent a therapeutic concentration of drug from accumulating in tumor cells. In antimicrotubular agents, such as the taxanes, additional mechanisms of tumor resistance can arise that prevent interaction with their target, β-tubulin.

Epothilones are naturally occurring macrolides that share a similar mechanism of action with taxanes. These agents induce microtubule polymerization at submicro-molar concentrations [14]. In the preclinical setting, epothilones possess potent antiproliferative activity in various tumor cell lines, particularly in the setting of taxane resistance [15-17]. Epothilones and paclitaxel compete for the same binding pocket on β-tubulin; however, epothilones and the taxanes bind to different sites on β-tubulin. Significantly, epothilones have low susceptibility to multiple mechanisms of tumor resistance, including overexpression of MDR-1, p-glycoprotein, and tubulin mutations [18-20]. The epothilones and their analogues therefore probably represent an important treatment option for patients with cancer, including those whose disease is resistant to other currently available treatments.

Currently, there are five epothilones being investigated in clinical trials: patupilone (epothilone B, EPO906), ixabepilone (aza-epothilone B, BMS-247550), BMS-310705 (a water-soluble semisynthetic analog of epothilone B), KOS-852 (epothilone D), and ZK-EPO [21]. Ixabepilone has been the most extensively studied and is the only epothilone approved by the US Food and Drug Administration for the treatment of cancer. Ixabepilone has a broad spectrum of activity against multiple cell lines and *in vivo* in animal models. Lee and colleagues tested ixabepilone in multiple human cancer cell lines, finding that in 18 out of 21 lines the half-maximal inhibitory concentration (IC_50_) values were 1.4 to 6 nM [20].

#### Clinical activity

To date, six relevant phase II clinical studies and one phase III clinical study have evaluated ixabepilone in patients with anthracycline-pretreated or taxane-pretreated or taxane-resistant advanced breast cancer (Table 1). Two phase II clinical studies in patients treated with anthra-cyclines revealed overall response rates (ORRs) of 41.5% [22] and 57% [23]. More importantly, in patients resistant to anthracyclines, taxanes, and/or capecitabine, single-agent ixabepilone showed an ORR of 11% in 113 evaluable patients, 50% of whom had stable disease (SD) [24]. Based on results from a pivotal phase III study [25], ixabepilone in combination with capecitabine was approved for the treatment of locally advanced breast cancer or MBC after failure of a taxane and an anthra-cycline. Ixabepilone monotherapy is indicated after failure of a taxane, anthracycline, and capecitabine, based on the results from the phase II study in this patient population [24].

**Table 1:** Clinical efficacy of ixabepilone in locally advanced and metastatic breast cancer

Author and reference	Trial design	Number of patients	Patient population	Dose schedule	ORR	PFS	Toxicity grade 3/4
Roche *et al.*[22]	Single arm, phase II	65	First-line MBC – prior adjuvant A (100%) and T (17%)	Ixa 40 mg/m^2^ every 3 weeks	41.5%	TTP 4.8 months (4.2 to 7.6), median OS 22 months (15.6 to 27)	Neutropenia 58%, PN 28%
Denduliri *et al.*[23]	Single arm, phase II	23	First-line MBC	Ixa 6 mg/m^2^/day on days 1 to 5 every 3 weeks	57%	TTP 5.5 months	Neutropenia 22%, fatigue 13%, nausea 9%
Perez *et al.*[24]	Single agent, phase II	126	Refractory to T, A, and CPC	Ixa 40 mg/m^2^ every 3 weeks	11.5% (95% CI: 6.3 to 18.9 months)	3.1 months (2.7 to 4.2 months)	Neutropenia 54%
Bunnell *et al.*[139]	Single arm, phase II	62	Refractory to A and T (100%)	Ixa 40 mg/m^2^ every 3 weeks plus CPC 1,000 mg/m^2^ twice daily for 14 days	30%	3.8 months (2.7 to 5.6 months)	Neutropenia 69%, HFS 34%, PN 19%
Thomas *et al.*[140]	Single arm, phase II	49	Second-line, third-line, or fourth-line	Ixa 40 mg/m^2^ every 3 weeks	12%	TTP 2.2 months, OS 7.9 months (6.1 to 14.5%)	Neutropenia 55%, PN 12.2%
*Low et al.*[141]	Single arm, phase II	37	First-line	Ixa 6 mg/m^2^/day on days 1 to 5 every 3 weeks	22% (9.8 to 38.2%)	TTP 2.6 months	Neutropenia 35%, FN 14%
Thomas *et al.*[25]	Randomized, phase III	752	>First-line	Ixa 40 mg/m^2^ every 3 weeks plus CPC 2,500 mg/m^2^ for 14 days vs. CPC 2,000 mg/m^2^ for 14 days	42% vs. 23%	5.3% vs. 3.8%	PN 23% vs. 0%, myalgias 8% vs. 0.3%, asthenia 7.8% vs. 0.8%

A, anthracyclines; CI, confidence interval; CPC, capecitabine; FN, febrile neutropenia; HFS, hand-and-foot syndrome; Ixa, ixabepilone; MBC, metastatic breast cancer; ORR, overall response rate; OS, overall survival; PFS, progression-free survival; PN, peripheral neuropathy; TTP, time to tumor progression; T, taxanes.

## Targeted therapy

### Anti-HER2 therapies: newer HER2-targeted agents

Epidermal growth factor receptor (EGFR) is a receptor tyrosine kinase frequently expressed in epithelial tumors. A wide variety of cellular functions are modulated by the four members of the EGFR family, which play a major role in promoting breast cancer cell proliferation and malignant growth [26]. EGFR is thus an attractive target for therapeutic intervention. This receptor family comprises four homologous receptors: EGFR (ErbB1/EGFR/ HER1), ErbB2 (HER2/*neu*)*,* ErbB3 (HER3), and ErbB4 (HER4). At least six different ligands, known as epidermal growth factor-like ligands, bind to EGFR [27]. After ligand binding, the ErbB receptor is activated by di-merization between two identical receptors (homo-dimerization) or between different receptors of the same family (heterodimerization) [28]. After receptor dimeri-zation, an activation cascade of multiple protein kinase activity and tyrosine autophosphorylation occurs, phos-phorylating several intracellular substrates including Ras-Raf-mitogen-activated protein kinase, phosphatidylinositol-3-kinase (PI3K)-Akt, and other important signaling that regulates apoptosis and cellular proliferation pathways [29,30].

In breast cancer, EGFR and HER2 are frequently over-expressed and are associated with aggressive clinical behavior and poor outcome [31,32]; however, the outcome for patients with these highly aggressive tumors has markedly improved with the development of anti-HER2 therapies. Trastuzumab is a recombinant humanized monoclonal antibody that binds with high affinity to the extracellular domain of HER2 and inhibits proliferation in human tumor cells that overexpress HER2 [33]. Trastuzumab was the first HER2-targeted therapy approved by the US Food and Drug Administration in 1998 for the treatment of HER2-overexpression MBC [34]. Several clinical trials subsequently established the fact that the addition of trastuzumab to adjuvant chemotherapy (either in sequence or in combination) resulted in significant improvements in disease-free and OS rates in patients with early-stage HER2-overexpression MBC [35-37]. Although trastuzumab represents the first success in targeted therapy for breast cancer, one-third of patients are resistant to the treatment and many questions remain about the mechanism of activity. Both antibody-mediated inhibition of HER2 and use of receptor tyrosine kinase inhibitors (TKIs) are proven beneficial strategies for tumors with HER2 overexpression.

Small-molecule TKIs compete with ATP for binding at the EGFR catalytic kinase domain, preventing signal transduction of both the Ras-RAF1-mitogen-activated protein kinase and PI3K pathways and leading to increased apoptosis and decreased cellular proliferation. These compounds may be reversible (that is, lapatinib, gefitinib, or erlotinib) or irreversible (carnetinib or neratinib). With the exception of gefitinib and erlotinib, which are considered pure EGFR inhibitors, the remaining TKIs are characterized by multiple kinase inhibitors [38]. The promiscuous nature of the multiple inhibitors has the potential to contribute to increased toxicity.

### Epidermal growth factor family inhibitors

#### Gefitinib

Gefitinib (formerly known as ZD1839) – a pure EFGR inhibitor – is a small-molecule anilinoquinazoline that reversibly inhibits EGFR tyrosine kinase autophosphorylation and inhibits downstream signaling [39]. In tumor cell lines, gefitinib inhibits the growth of cells that express high levels of EGFR. Gefitinib has been shown to block EGFR downstream signal transduction pathways; this induces cell cycle arrest, which leads to accumulation of the cyclin-dependent kinase 2 inhibitor p27^KIP1^ and marked accumulation of hypophosphorylated Rb protein, which leads to G1 arrest [40].

Multiple phase I and phase II studies using gefitinib as a single agent or combined with chemotherapy in breast cancer patients have been completed. Gefitinib as a single agent resulted in minimal clinical benefit (CB), and the nonrandomized combination studies showed that gefitinib did not significantly increase disease-free survival or ORR. A preliminary exploratory analysis of two randomized, phase II, placebo-controlled trials comparing anas-trozole or tamoxifen with or without gefitinib was published [41]. In both trials, endocrine therapy-naïve patients experienced prolonged progression-free survival (PFS) with hormone therapy plus gefitinib.

#### Erlotinib

Erlotinib (formerly known as OSI-774) – a pure EFGR inhibitor – is a small-molecule quinazolinamine that reversibly inhibits EGFR tyrosine kinase and prevents receptor autophosphorylation [42]. Several trials of erlotinib in combination with drugs known to be active in breast cancer were recently conducted. In a dose-escalation study of erlotinib in combination with capecitabine and docetaxel in patients with MBC, two patients had complete response and 12 patients had partial response (PR) (ORR, 67%) [43]. The main toxic effects of the regimen consisted of skin and gastrointestinal manifestations.

Several other preliminary studies combining erlotinib with docetaxel [44], with vinorelbine plus capecitabine [45], and with bevacizumab [46] have been reported.

On the basis of data from a preclinical mouse xenograft model, a clinical trial was conducted involving patients with operable breast cancer stage I to stage IIIA. Fifty-two patients received erlotinib at 150 mg/day orally for 6 to 14 days before surgery [47]. A reduction in Ki67 expression, a surrogate marker of proliferation, was demonstrated in ER-positive tumors but not in those that overexpressed HER2 or in those with triple receptor-negative (TRN) breast cancer.

#### Trastuzumab-DM1

Trastuzumab–DM1 was the first antibody-drug conjugate based on trastuzumab, which consists of trastuzumab linked to an antimicrotubule drug, maytansine (also known as DM1). The potential advantage of this conjugate is that trastuzumab targets DM1 specifically into tumor tissues, which may reduce toxicity.

In addition, trastuzumab has its own anticancer activity. Trastuzumab-DM1 showed activity in a xenograft model of HER2-positive, trastuzumab-resistant tumors [48]. A phase I study of trastuzumab-DM1 in heavily pretreated patients with HER2-overexpressing MBC showed clinical activity, with thrombocytopenia as the dose-limiting toxicity (DLT), at a dosage of 4.8 mg/kg every 3 weeks. The recommended dosage for phase II studies was 3.6 mg/kg every 3 weeks [49]. A recent preliminary report of a phase II study of trastuzumab-DM1 in 112 patients with HER2-overexpressing MBC in whom treatment with trastuzumab, lapatinib, or both had failed showed promising activity, with an independent review panel confirming an ORR of 25% (28 patients) and a CB rate of 34% (38 patients) [50].

Two phase III studies of trastuzumab–DM1 are ongoing. One trial is testing the activity of trastuzumab–DM1 versus standard therapy with lapatinib–capecitabine as the second-line therapy for patients with HER2-positive MBC. The other ongoing study is testing docetaxel plus trastuzumab versus single-agent trastuzumab–DM1 as the first-line therapy for HER2-positive MBC.

#### Cetuximab

Cetuximab (formerly known as C225) is a recombinant chimeric human murine IgG1 antibody that binds to the extracellular domain of the EGFR [51]. Cetuximab was approved for use in patients with EGFR-expressing metastatic colorectal cancer refractory to irinotecan-based chemotherapy [52]. A phase I dose-escalation study of cetuximab and paclitaxel in patients with MBC showed that two out of six patients in the second cohort (cetuximab at 100 mg/m^2^) developed DLT effects in the form of grade 3 rash. Ten patients were evaluable for response; two of them experienced SD, and eight had progressive disease [53].

Preliminary results were reported from a randomized trial in which patients with TRN MBC refractory to between one and three lines of chemotherapy were randomly assigned to carboplatin plus cetuximab versus cetuximab alone [54]. Cetuximab alone was well tolerated, with a very modest ORR of 6%. The carboplatin plus cetuximab combination arm achieved an ORR of 18% and CB of 27%.

A preliminary report in patients with MBC treated with irinotecan plus carboplatin versus the same regimen plus cetuximab showed that cetuximab did not improve antitumor activity, PFS, or OS, but did increase toxicity [55]. On subset analysis, however, the addition of cetuximab increased the ORR associated with irinotecan plus carboplatin in TRN breast cancer.

### Dual EGFR and HER2 inhibitors

#### Lapatinib

Lapatinib (formerly known as GW572016) is currently the most advanced oral selective dual-EGFR/HER2 reversible inhibitor in terms of clinical development in breast cancer. The rationale for developing this dual EGFR/HER2 TKI was to sustain synergistic inhibition of cancer cells by simultaneously targeting receptors in both cell lines, resulting in more potent inhibition in cell growth than could be achieved by targeting either EGFR or HER2 alone [56]. One important characteristic of lapatinib, compared with other selective EGFR TKIs such as erlotinib and gefitinib, is a slower dissociation rate from EGFR, resulting in a prolonged effect at the downregulated target site [57]. In tumor cell lines and xenograft models, lapatinib has inhibited EGFR and p-ErbB2, p-Erk1/2, p-AKT, and cyclin D [58,59], and this effect was dose and time dependent.

In a phase II study, 229 patients with HER2-amplified (*n =* 140) or HER2-negative (*n =* 89) triple-refractory disease (to anthracyclines, taxanes, and capecitabine) received lapatinib at 1,500 mg/day as monotherapy [60]. Patients with HER2 amplification had a 4.3% ORR by investigator review and a 1.4% ORR by independent review. The median PFS was similar in both patient groups, and 6% of HER2-amplified patients derived CB from lapatinib. Grade 3 and grade 4 toxic effects included diarrhea (54%), rash (30%), and nausea (24%). In this group of heavily pretreated patients (76% of whom received four or more lines of prior therapy), lapatinib had modest activity in HER2-overexpressed MBC.

##### Lapatinib in metastatic breast cancer

Lapatinib was approved by the US Food and Drug Administration in 2007 for use in combination with capecitabine for treatment of HER2-overexpressed MBC that had progressed with standard treatment [61]. The study was designated to compare time to tumor progression between two arms, and the secondary end point was OS. Patients were randomly assigned to receive either lapatinib (1,250 mg/day orally for 14 days, followed by 1 week of rest) or a combination of lapatinib and capecitabine (2,000 mg/m^2^/day orally for 14 days, followed by 1 week of rest).

The study was closed prematurely after the first interim analysis, when 321 patients had been accrued, because results showed that the addition of lapatinib to capecitabine was associated with a 51% risk reduction of disease progression (HR, 0.49; 95% CI, 0.34 to 0.71; *P* <0.001). The median time to tumor progression was 8.4 months for the combination arm and 4.4 months for the monotherapy arm. The ORR was 22.5% for the combination arm versus 14.3% for the monotherapy arm (*P =* 0.113). Toxic effects were similar in both arms. The most common adverse effects for the combination versus the monotherapy were diarrhea (58% vs. 38%), hand-foot syndrome (43% vs. 34%), and rash (34.5% vs. 30%). In the monotherapy group 11 women had progressive central nervous system metastasis, compared with four women in the combination therapy group. This difference was not statistically significant (*P =* 0.10). Cardiotoxicity was observed in the combination arm: four patients experienced cardiac events related to treatment and fully recovered. In the capecitabine monotherapy group, one patient experienced a cardiac event unrelated to treatment, which remained unresolved.

##### Lapatinib in combination with trastuzumab

A preclinical study demonstrated synergistic interaction between trastuzumab and lapatinib in HER2-overexpressed breast cancer cells lines and tumor xenografts [62]. A preliminary report presented at the American Society of Clinical Oncology 2008 Annual Meeting revealed that the addition of trastuzumab to lapatinib, compared with lapatinib alone, significantly improved PFS and CB [63]. The large, ongoing Aphrodite trial has a target population of 8,000 patients with HER2-overexpressed MBC. In this four-treatment-arm randomized trial, both trastuzumab and lapatinib were combined in the adjuvant setting. The control arm received standard trastuzumab for 1 year, and each of the three experimental arms received one of the following for 1 year: lapatinib, sequencing trastuzumab and lapatinib, or combined trastuzumab and lapatinib.

##### Lapatinib in combination with hormonal agents

Accumulating evidence is showing that signaling interplay occurs between the ER, HER2, EGFR, and IFG-1 receptors, affecting acquired resistance to hormonal therapies [64,65]. In a preclinical study, Chu and colleagues demonstrated that lapatinib can restore tamoxifen sensitivity in ER-positive, tamoxifen-resistant breast cancer models [66]. In a phase III study of letrozole with or without lapatinib in postmenopausal patients with hormone-sensitive, HER2-positive MBC, the combination resulted in improved PFS, from 3.0 to 8.2 months [67]. The ongoing LET-LOB study (Letrozole with Lapatinib) is a European phase II clinical trial of letrozole with or without lapatinib as neoadjuvant treatment in hormone-sensitive, HER2-negative operable breast cancer [68].

##### Lapatinib in the neoadjuvant setting

The Neo-ALTTO trial is a randomized, open-labeled, multicentric, phase III study comparing the efficacy of neoadjuvant lapatinib plus paclitaxel with that of trastuzumab plus paclitaxel and with concomitant lapatinib and trastuzumab plus paclitaxel given as neoadjuvant treatment in HER2-overexpressed operable breast cancer with a tumor diameter >2 cm [69]. Preliminary results are pending.

### Other HER2-directed tyrosine kinase inhibitors

#### Neratinib

Neratinib (formerly known as HKI-272) is the next most advanced agent in clinical development after lapatinib. Neratinib is an irreversible inhibitor of HER2 and EGFR with IC_50_ values of 59 nM and 92 nM, respectively [70]. In a phase I trial in solid tumors, the maximum tolerated dose was 320 mg and the DLT was grade 3 diarrhea [71]. Preliminary findings from a phase II study evaluating neratinib at a dose of 240 mg/day in patients with HER2-amplified trastuzumab-naïve or previously treated locally advanced breast cancer or MBC showed that patients in the previously treated group (*n =* 61) had an ORR of 26% with a median PFS of 23 weeks [72]. In the trastuzumab-naïve cohort, the ORR was 77% with a median PFS of 16 weeks. Diarrhea was the most common adverse event, and was present in 93% of patients with grade 3 toxicity and in 21% of patients with grade 4 toxicity.

Neratinib was combined with paclitaxel in advanced, previously treated HER2-positive MBC [73]. No DLT was encountered, and five out of 35 patients had PR. The most common grade 3 and grade 4 toxicities were diarrhea (20%), neutropenia (9%), and dehydration (2%).

Neratinib and trastuzumab exert their effect on the HER2 receptor at various molecular sites, and it has been suggested that the combination of both agents may be synergistic. In a phase I/II study in patients with advanced HER2-positive breast cancer that had progressed after trastuzumab therapy, patients received 240 mg neratinib with standard doses of trastuzumab [74]. The ORR was 27%, which included 7% complete responses. The 16-week PFS rate was 45%, and the median PFS duration was 19 weeks. No DLTs were observed, and diarrhea, nausea, anorexia, and vomiting were the most frequent adverse events.

Currently, three large phase III studies using neratinib are ongoing. A phase III, randomized study (NCT00777101) comparing neratinib with a combination of capecitabine and lapatinib in locally advanced breast cancer or MBC with HER2 amplification is under way. The primary objective of this study is to compare PFS in two regimens. Neratinib is also being compared with placebo in a phase III study of early-stage HER2-overexpressed breast cancer in patients who have been treated with trastuzumab (NCT00878709). Finally, a combination of neratinib plus paclitaxel is being compared with trastuzumab plus paclitaxel for the first-line treatment of HER2-positive locally advanced breast cancer or MBC (NCT00915018).

#### Canertinib

Canertinib (formerly known as CI-1033) is a small-molecule TKI that potently inhibits all active members of the EGFR family. One important characteristic of canertinib is its property of irreversible inhibition through the ERB receptor, achieved by covalently modifying a cysteine residue in the ATP-binding site. Interestingly, this property determines canertinib’s ability to induce ubiquitylation and degradation of both ErbB1 and ErbB2 [75], a property not shared by reversible TKIs.

In a phase I multicenter study [76], 32 patients with advanced solid malignancies received a starting dose of canertinib at 300 mg/day; at a dose of 560 mg/day, grade 3 DLT was observed in three of these patients. The maximum tolerated dose was declared at 450 mg, at which level one out of six patients experienced grade 3 dehydration associated with grade 2 stomatitis. Overall, gastrointestinal and skin toxicity were the most frequently reported adverse events. Efficacy analysis showed no objective response in 15 patients with measurable disease, and six patients had SD. Marked interpatient variability was found in 22 patients in the pharmacokinetic data, apparently not associated with the drug concentration in the plasma.

#### EKB-569

EKB-569 has a molecule structure similar to that of neratinib. It is a potent inhibitor of EGFR with an IC_50_ of 39 nM in an autophosphorylation assay, where it was substantially less active toward HER2 with an IC_50_ of 1,255 nM [77]*.* Results from a recent phase I dose-escalation study using two different dose schedules have been reported [78]. Thirty patients were treated daily for 14 days of a 28-day cycle, and 29 patients received continuous daily dosing. The DLT was grade 3 diarrhea, and the maximum tolerated dose was 75 mg/day. There were no objective responses, although 24 patients had SD for 8 weeks.

#### Pertuzumab

Pertuzumab is the first in a new class of agents known as HER dimerization inhibitors. Pertuzumab binds to HER2, the most common HER pairing partner, at the dimerization domain [79], inhibiting its ability to form dimers with other HER receptors [80,81]. The original findings from pertuzumab treatment in patients with solid tumors included good tolerance and clinical activity and supported a 3-week dosing schedule [82]. Interestingly, the pertuzumab binding site within domain II does not overlap with the epitope on HER2 that is recognized by trastuzumab, which allows combined targeting of both monoclonal antibodies against HER2. Preclinical data from studies combining pertuzumab and trastuzumab have shown that these two agents synergistically inhibit the survival of breast tumor cells [83]. The CB of this combination has been reported in patients with HER2 overexpression [84].

In preliminary findings from a phase II study of combined trastuzumab and pertuzumab in patients with HER2-overexpressed MBC, a 40% CB rate with multiple complete responses and PRs was described [85].

#### Ertumaxomab

Ertumaxomab is a trifunctional bispecific antibody – targeting HER2 on tumor cells and CD3 on T cells – that can redirect T cells, macrophages, dendritic cells, and natural killer cells to the sites of tumor metastases [86,87].

## Antiangiogenic therapy: current and novel therapies

Substantial preclinical and indirect clinical evidence suggests that angiogenesis plays an essential role in breast cancer development, invasion, and metastasis [88]. Angiogenesis is a fundamental mechanism in biology in which new blood vessels are formed from existing vasculature during a complex multistep process that is tightly regulated by proangiogenic factors and involves autocrine and paracrine signaling. Since VEGF is essential for the development of neovasculature at very early stages of tumorigenesis, it is believed to play a key role in the formation of tumor metastasis. The transition of a tumor from the avascular or prevascular phase to the vascular phase (increased growth and metastatic potential) is termed the angiogenic switch [89]. This switch – which is considered a hallmark of the malignancy process – is believed to be stimulated by increased expression of proangiogenic factors such as VEGF, basic fibroblast growth factor, and transforming growth factor β, and by decreased expression of antiangiogenic factors such as IFNα or thrompospondin-1 [90].

The VEGF-related gene family comprises six secreted glycoproteins: VEGF-A, VEGF-B, VEGF-C, VEGF-D, VEGF-E, placenta growth factor-1 and placenta growth factor-2 [91]. The past decade has witnessed major advances in the development of therapeutic agents that modulate tumor angiogenesis. Some of these agents have been shown to be effective in inhibiting tumor angiogenesis and have become an important part of standard cancer treatment: bevacizumab in colon, lung, breast, and renal cell carcinoma; sorafenib in renal cell carcinoma and hepatocellular carcinoma; and sunitinib in renal cell carcinoma and gastrointestinal stromal tumors.

### Bevacizumab

Bevacizumab is derived from the murine VEGF monoclonal antibody A4.6.1 [92] and is composed of ~93% human and ~7% murine protein sequences. Experimental studies have showed that bevacizumab neutralizes all isoforms of human VEGF with a dissociation constant of 1.1 nmol/l [93]. Clinical pharmacology studies of bevacizumab have demonstrated a linear pharmacokinetics profile and a long terminal half-life of approximately 21 days (range, 11 to 50 days).

#### Phase I/II studies of bevacizumab as a single agent and combined with chemotherapy

Two phase I clinical trials of bevacizumab as a single agent in solid tumors have been reported. In the first trial, 25 patients with refractory solid tumors received doses of bevacizumab ranging from 0.1 to 10 mg/kg over 8 weeks [94]. In the second trial, bevacizumab was administered to 12 patients at a dose of 3 mg/kg in combination with chemotherapy [95]. These studies showed that bevacizumab is safe and without DLTs at doses up to 10 mg/kg and can be combined with chemotherapy, apparently without synergistic toxicity.

An early dose-escalation phase I/II clinical trial was conducted in 75 patients with MBC who were treated with bevacizumab to determine the agent’s safety, efficacy, and pharmacokinetic characteristics [96]. Most of the patients (96%) had received prior anthracycline-based or taxane-based chemotherapy for metastatic disease, and 28% of patients were HER2-positive. There were three different dose escalations at 3 mg/kg, 10 mg/kg, and 20 mg/kg every 2 weeks. The ORR was 9.3% (confirmed response rate of 6.7%). The median duration of confirmed response was 5.5 months (range, 2.3 to 13.7 months). Four patients (5.3%) discontinued the study treatment because of an adverse event. Hypertension was reported as an adverse event in 22% of patients. The optimal dose of bevacizumab in this trial was thus 10 mg/kg every other week, and toxicity was deemed to be acceptable.

#### Phase III studies of bevacizumab in previously treated MBC

Based on previous data, a phase III randomized trial was undertaken to evaluate bevacizumab treatment in women with heavily pretreated MBC [97]. In these patients, MBC had been previously refractory to anthracyclines and taxanes and had relapsed within the first 12 months of patients’ completion of adjuvant therapy. A total of 462 patients were randomized to receive bevacizumab at 15 mg/kg every 3 weeks plus capecitabine at 2,500 mg/m^2^ in two divided doses for 2 weeks out of every 3 weeks, or capecitabine alone. The primary end point of the trial was PFS and was statistically identical between both arms (capecitabine, 4.2 months vs. capecitabine plus bevacizumab, 4.9 months). The ORR was significantly higher in the combination arm (19.8%) than in the single-agent (capecitabine) arm (9.1%; *P =* 0.001). The responses to bevacizumab tended to be short and were not translated into improved PFS duration, which was 4.9 months in the combination arm and 4.2 months in the single-agent (capecitabine) arm.

#### Phase III study of bevacizumab as first-line treatment for MBC

**Figure 1:**
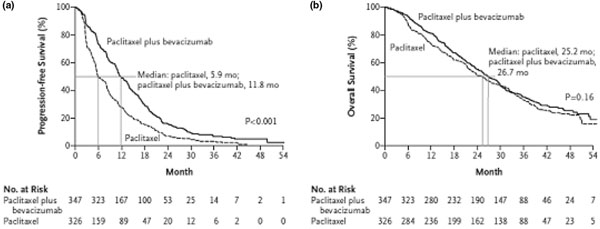
**Eastern Cooperative Oncology Group 2100 trial.** Phase III study of paclitaxel with or without bevacizumab. **(a)** Progression-free survival. **(b)** Overall survival. mo, months.

The Eastern Cooperative Oncology Group 2100 trial enrolled 680 patients with previously untreated locally recurrent breast cancer or MBC [98]. Patients received weekly paclitaxel at 90 mg/m^2^ on days 1, 8, and 15, with or without bevacizumab at 10 mg/kg on days 1 and 15, in 4-week cycles until disease progression. All patients with HER2-positive disease were required to have received prior trastuzumab, and most (96%) were HER2-negative. The primary end point of the study was PFS, which was significantly improved in patients who received the combination of bevacizumab plus paclitaxel versus single-agent paclitaxel (11.8 vs. 5.9 months; HR, 0.60; 95% CI, 0.43 to 0.62; *P* ≤0.001) (Figure 1). The PFS benefit with bevacizumab was observed across all subgroups, regardless of age, number of metastatic sites, previous adjuvant taxane use, disease-free interval after adjuvant therapy, and hormone receptor status. The ORR was 36.9% in the combination arm versus 21.2% in the single-agent paclitaxel arm (*P* <0.00l). The safety profile of bevacizumab in the Eastern Cooperative Oncology Group 2100 trial, as reported in *The New England Journal of Medicine*[98], showed no increase in deaths; however, the trial was audited by a group of experts who found several cases of small-bowel perforation that the investigators had not attributed to bevacizumab. On 22 February 2008, the US Food and Drug Administration approved bevacizumab in combination with paclitaxel as first-line chemotherapy in patients with refractory MBC. A final OS report from the AVADO trial – a phase III placebo-controlled, randomized study of two doses of bevacizumab with or without docetaxel as first-line therapy for patients with recurrent or MBC – was presented at the San Antonio Breast Cancer Symposium (SABCS) 2009 [99]. An increase in PFS with docetaxel (100 mg/m^2^ every 3 weeks) plus bevacizumab (7.5 mg/kg or 15 mg/kg every week) was observed. In 736 patients, the drugs were analyzed for toxicity and efficacy. In terms of primary objective, the HR for docetaxel plus bevacizumab at 7.5 mg/kg was 0.80 (95% CI, 0.65 to 1.00; *P =* 0.045) and for docetaxel plus bevacizumab at 15 mg/kg was 0.67 (95% CI, 0.48 to 0.78; *P =* 0.0002). The ORR was 46.4% for docetaxel and placebo, 55.2% for docetaxel and bevacizumab at 7.5 mg/kg, and 64.1% for docetaxel and bevacizumab at 15 mg/kg. Grade 3 and grade 4 adverse events were 67% for docetaxel and placebo, 74.8% for docetaxel and bevacizumab at 7.5 mg/kg, and 74.1% for docetaxel and bevacizumab at 15 mg/kg.

#### Bevacizumab in combination with other targeted therapies

A recent phase II clinical trial combined erlotinib and bevacizumab in patients with MBC who had received one or two prior chemotherapy regimens [100]. Thirty-eight patients were treated with erlotinib (150 mg/day orally) and bevacizumab (15 mg/kg intravenously every 3 weeks), and the primary end point was the response rate per Response Evaluation Criteria in Solid Tumors. Patients received a median of three cycles of treatment (range, 1 to 85 cycles). One patient (3%) had PR after three cycles of therapy, and 15 patients (40%) had SD at 9 weeks. The most common adverse events for the 38 patients were diarrhea 84% (grade 3 in only 3%), rash 76% (grades 1 and 2 only), and fatigue 63% (grades 1 and 2 only). Four patients (11%) developed grade 3 hypertension that was controlled by oral medication, and eight patients (21%) developed proteinuria. There were two grade 4 events: thrombosis and myalgias. Twenty-five patients were negative for EGFR tyrosine-kinase domain mutational analysis, and the level of EGFR expression was not predictive of response to therapy.

Mature data from five studies revealed improvement in PFS when bevacizumab was added to standard chemotherapy [98,99,101] (Table 2). The RIBBON-2 study became the first positive phase III study of bevacizumab in second-line MBC. Bevacizumab is currently being explored for use in early breast cancer, as neoadjuvant treatment in the NSABP B40 study, in TRN breast cancer (BEATRICE study), and in HER2-positive disease as adjuvant treatment (BETH study).

**Table 2:** Phase III clinical studies incorporating bevacizumab to chemotherapy in breast cancer patients

Trial and reference	Number of patients	Patient population	Bevacizumab dose	Combination therapy	End point	Benefit in anti-VEGF therapy	Study primary results
AVF2119[97]	462	Pretreated MBC	15 mg/kg every 3 weeks	Cap 2,500 mg/ m^2^/day from days 1 to 14	PFS	**No**	Bev and Cap significantly increased the ORR compared with single-agent Cap (9.1% vs. 19.8%, *P =* 0.001), but not PFS (4.2 vs. 4.0 months; HR, 0.98). No significant differences were found in incidence of diarrhea, hand-foot syndrome, and serious bleeding episodes between treatment groups
ECOG 2100 [98]	722	First-line MBC	10 mg/kg every 2 weeks	P 90 mg/m^2^ days 1,8,15	PFS	Yes	Bev and P significantly prolonged PFS compared with P alone (median, 11.8 vs. 5.9 months; HR for progression 0.60, *P* <0.001) and increased ORR (36.9% vs. 21.2%). No differences in OS between two groups (median 26.7 vs. 25.5 months; HR 0.88, *P* = 0.16). Adverse effects: grade 3 or 4 hypertension (14.8% vs. 0%, *P* <0.001), proteinuria (3.6% vs. 0%, *P* <0.001), headache (2.2% vs. 0%, *P* = 0.008) and cerebrovascular ischemia (1.9% vs. 0%, *P* = 0.02) were more common in patients receiving combination treatment
AVADO [99]	736	First-line MBC	7.5 mg/kg every 3 weeks	D 100 mg/m^2^ every 3 weeks	PFS	Yes	In stratified analysis, patients receiving Bev had significantly longer PFS compared with the D monotherapy group (Bev at 7.5 mg/kg: me-dian PFS 8.7 vs. 8.0 months, HR 0.79, *P* = 0.0318; Bev at 15 mg/kg: median PFS 8.8 vs. 8.0 months, HR 0.72, *P* = 0.0099). ORR improved with the addition of Bev (Bev 7.5 mg/kg: 55% vs. 44%, *P* = 0.0295; Bev 15 mg/kg: 63% vs. 44%, *P* = 0.0001). The study was not powered to find differences in OS
			15 mg/kg every 3 weeks				
RIBBON-1 [101]	1,237^a^	First-line MBC	15 mg/kg every 3 weeks	Cap, taxanes (Nab-Pac and D), anthracycline	PFS	Yes	The median follow-up was 15.6 months in the Cap cohort and 19.2 months in the taxanes and anthracycline cohort. The addition of Bev to Cap, taxanes, or anthracycline-based chemotherapy resulted in statistically significant improvement in PFS
RIBBON-2 [102]	684	Second-line MBC	15 mg/kg every 3 weeks	Cap, taxanes (Nab-Pac and D), anthracycline, Cap, gemcitabine, vinorelbine	PFS	Yes	Median PFS with Bev was 7.2 vs. 5.1 months (HR 0.78, *P* = 0.0072). A trend for higher objective response rate with Bev 39.5% vs. 29.6%; *P* = 0.013, not significant at prespecified 0.01. No difference in OS with combination therapy compared with chemotherapy alone (18 vs. 16.4 months; HR 0.90, *P* = 0.3741). Among the different chemotherapy regimens used in the trial, taxanes and Cap appeared to be more effective, whereas gemcitabine and vinorelbine appeared less effective
MO19391 [103]	2.027^a^	HER2^-^ MBC or HER2^+^ if previous Tz	10 mg/kg every 2 weeks or 15 mg/kg every 3 weeks	Taxane-based chemotherapy	Safety	Yes	Median follow-up was 7.4 months. ~75% of patients received taxanes, and 25% were treated with nontaxane regimens (Cap and vinorelbine). Safety and efficacy of Bev plus D or P was similar to results of the E2100 and AVADO trials

Bev, bevacizumab; Cap, capecitabine; D, docetaxel; HER2, human epidermal growth factor receptor 2; HR, hazard ratio; MBC, metastatic breast cancer; Nab-Pac, Nab-paclitaxel; ORR, overall response rate; OS, overall survival; PFS, progression-free survival; P, paclitaxel; Tz, trastu-zumab; VEGF, vascular endothelial growth factor.

^a^Currently enrolling patients.

## Emerging anti-VEGF therapies

### Tyrosine kinase inhibitors

#### Sunitinib

Sunitinib malate (formerly known as SU1128) is an oral TKI that targets several receptor tyrosine kinases, including VEGF receptor (VEGFR-1, VEGFR-2, and VEGFR-3), platelet-derived growth factor receptor (PDGFR-α and PDGFR-β), cKIT, and colony-stimulator factor 1 receptor [102]. In preclinical models, sunitinib administration resulted in significant reduction in phos-photyrosine levels of VEGFR-2, PDGFR-β, and KIT, which correlated with tumor growth inhibition. Treatment with sunitinib at 40 to 80 mg/kg/day (orally) displayed potent and broad-spectrum antitumor activity in mouse xenograft models, and resulted in inhibited growth in several human cell lines, including the breast cancer cell line MDA-MB-435 [103].

In one of the first studies of sunitinib in patients with solid tumors, the pharmacokinetic characteristics and safety were evaluated. A total of 28 patients received sunitinib orally for 4 weeks at doses ranging from 15 to 59 mg/m^2^/day (50 mg every other day to 150 mg/day) [104].

Sunitinib was subsequently evaluated in a multicentric phase II trial in patients with MBC previously treated with anthracyclines and taxanes [105]. The primary trial objective was to determine the antitumor activity of sunitinib, starting at a dose of 50 mg administered once daily for 4 weeks followed by 2 weeks off treatment, in repeated 6-week cycles. Sixty-four patients were included in the study; seven patients (11%) achieved PR with a median duration of 19 weeks, and three patients (5%) had SD for 6 months, yielding a CB rate of 16%. The median duration of response was 19 weeks, and the median time to tumor progression was 10 weeks. The overall probability of survival at 1 year was 41% (95% CI, 28 to 54%), and the median OS was 38 weeks (95% CI, 28 to 63 weeks). Notably, responses occurred in three out of 20 patients (15%) with TRN MBC, and in three out of 12 patients (25%) with HER2-positive tumors. One-third of patients experienced grade 3 neutropenia, and all hematologic abnormalities resolved rapidly during off-treatment periods.

A preliminary report described the results of sunitinib combined with metronomic dosing of cyclophosphamide and methotrexate in patients with advanced breast cancer [106]. A total 15 patients were treated in three dose cohorts of sunitinib (12.5 mg/day, 25.0 mg/day, and 37.5 mg/day). Three patients developed grade 3 neutropenia and five patients developed mucositis. One patient had PR at week 14, and one patient had SD for 47 weeks. Enrollment for this study continues.

Findings from a preliminary report of a phase III study comparing sunitinib with capecitabine in previously treated HER2-negative MBC were recently presented at the SABCS 2009 [107]. A total of 482 patients had been randomized 1:1 to sunitinib (37.5 mg/day orally) and capecitabine (1,000 to 1,250 mg/m^2^/day orally from day 1 to 14), and the primary end point was PFS. The ORR and CB for patients treated with sunitinib were 11.3% and 19.3%, and for those treated with capecitabine were 16.4% and 27%, respectively (odds ratio, 0.65; 95% CI, 0.4 to 1.1). The PFS for patients treated with sunitinib and capecitabine was 2.8 months and 4.2 months, respectively (HR, 1.47; 95% CI, 1.16 to 1.87; *P =* 0.002), and the OS duration was 15.3 months and 24.6 months, respectively (HR, 1.17; 95% CI, 0.84 to 1.63; *P =* 0.350).

One randomized phase III trial (SUN 1094) that included sunitinib as the experimental arm was recently closed because the primary end point could not be met. This study compared sunitinib plus paclitaxel with bevacizumab plus paclitaxel as a first-line metastatic regimen. Two large ongoing phase III trials are comparing sunitinib plus docetaxel with docetaxel (SUN 1064) in first-line MBC and comparing sunitinib plus capecitabine with capecitabine (SUN 1099) in second-line MBC.

#### Sorafenib

The developers of sorafenib (formerly known as BAY43-9006) have mainly focused on improving its activity against Raf-1 kinase, which has an IC_50_of 12 nM both *in vitro* and *in vivo*[108]. Sorafenib has been evaluated in multiple phase I trials of refractory solid tumors. All trials identified hand-foot syndrome as the prominent DLT. Multiple trials using sorafenib in combination with chemotherapy have been reported [109]. For example, a two-stage, phase II, single-agent study in patients with MBC refractory to anthracyclines and taxanes was reported in which the initial dose was 400 mg sorafenib twice daily and the primary end point was ORR [110]. Among 20 patients eligible for analysis of efficacy, one patient (5%) achieved PR for 3.6 months. Because of a lack of sufficient response, the study was closed without proceeding to the second stage or accrual.

Two recent preliminary reports were presented at the SABCS 2009. In one randomized phase IIb study, which included 229 patients with locally advanced breast cancer or MBC, patients had been treated with sorafenib plus capecitabine versus capecitabine (SOLTI-0701) [107]. In the combination arm, the median PFS increased from 4.1 to 6.4 months (HR, 0.576; *P =* 0.0006). These results represent a 42% reduction in the risk of disease progression or death. The ORR for the combination of sorafenib plus capecitabine was 38% and for capecitabine plus placebo was 31% (*P =* 0.1229). Adverse events were significantly higher in the combination arm, with hand-foot syndrome grade 3 seen in 45% versus 13% in the capecitabine plus placebo arm.

An international phase IIb study randomized 220 patients with locally advanced breast cancer or MBC to sorafenib plus paclitaxel versus paclitaxel as the first-line treatment [111]. Approximately, three-quarters of patients were accrued in India and 20% in the United States. Patients treated with the sorafenib and paclitaxel combination had a longer PFS (6.9 months) than did those in the single-agent paclitaxel arm (5.6 months) (HR, 0.788; 95% CI, 0.558 to 1.112; *P =* 0.0857). The ORR for patients treated with the combination or single-agent paclitaxel was 67% and 54%, respectively (*P =* 0.023). Grade 3 adverse effects for hand-foot syndrome were 30% and 3% for the combination arm and single-agent paclitaxel, respectively. There was significant imbalance in regional patient characteristics with reference to age, hormone status, and prior chemotherapy, which made extracting solid conclusions from this trial difficult.

#### Motesanib

Motesanib (formerly known as AMG 706) is an orally administered multiple TKI of VEGF, platelet-derived growth factor, and KIT. Preclinical activity has been documented in multiple breast cancer cell lines. A 10-month analysis from the CIRG/TORI 010 trial was presented at the SABCS 2009 [112]. A total of 282 patients were randomized to one of three arms: motesanib plus paclitaxel, paclitaxel plus placebo, or paclitaxel plus bevacizumab. Patients were treated until progressive disease or intolerable toxicity, and the primary end point was ORR. Patients treated with motesanib plus paclitaxel had an ORR of 49.5%, compared with 51.55% for patients treated with paclitaxel plus bevacizumab. The PFS was 9.49 months (range, 8.41 to 12.1 months) for the motesanib plus paclitaxel arm and 11.5 months (range, 9.3 to 15.4 months) for the paclitaxel plus bevacizumab arm. Hepatobiliary toxicity seen with motesanib emerged as a unique toxicity with an unknown etiology. Eight out of 92 patients in the motesanib arm (8.6%) experienced grade 3 to 5 toxicity, including cholecystitis, gallbladder enlargement, cholestasis, and jaundice.

#### Vandetanib

Vandetanib (formerly known as ZD6474) inhibits two key pathways in tumor growth: VEGFR-dependent tumor angiogenesis, and EGFR-dependent tumor cell proliferation and survival. This compound is a potent inhibitor of kinase insert domain-contained receptor VEGFR-2 (IC_50_ = 40 nM), VEGFR-3 (IC_50_ = 110 nM), and EGFR/ HER1 (IC_50_ = 500 nM) [113]. Preclinical data have shown that the inhibition of EGFR signaling can inhibit the secretion of VEGF, as well as other proangiogenic factors such as basic fibroblast growth factor and transforming growth factor *α*[114]. The antitumor activity of vandetanib against EGFR may therefore reduce the levels of VEGF and other growth factors released by tumor cells. In a very elegant publication, Mi and Lou showed that vandetanib reversed p-glycoprotein-mediated multidrug resistance to Adriamycin, docetaxel, and vinorelbine in two p-glycoprotein-overexpressed breast cancer cell lines derived from MCF-7/Adriamycin and KBV200 [115]. In addition, this study suggested that vandetanib is not a substrate of p-glycoprotein.

In a phase I dose-escalation study of vandetanib in *77* patients with solid tumors [116], patients received once-daily oral vandetanib (50 to 600 mg daily) in 28-day cycles until disease progression or unacceptable toxicity. Pharmacokinetic analysis revealed a half-life of ~120 hours with significant interpatient variability. The study established that a dose of 300 mg daily was well tolerated, and the most common DLTs were diarrhea, hypertension, and rash. Asymptomatic prolongation of the QT interval corrected for heart rate was more frequent with doses >500 mg daily.

In a phase II trial, Miller and colleagues treated 46 patients with MBC refractory to taxanes and anthracyclines [111]. The primary end point was ORR. The authors used a pharmacokinetic analysis from a previous phase I study that suggested potentially therapeutic levels of vandetanib would be achieved with both the 100 mg and 300 mg doses. Two patient cohorts were designated in this trial: those who initially received 100 mg daily, and those enrolled later who received 300 mg daily in the absence of grade 3 or 4 prolongation of the QT interval corrected for heart rate. Forty-four patients who were evaluable for drug efficacy had no objective responses, and one patient had SD for longer than 24 months. The authors hypothesized that the lack of activity could be related to an inadequate blood concentration of vandetanib, although most patients achieved plasma concentration above the IC_50_; however, the common toxic effects for VEGF inhibitors (for example, hypertension, headache, and thrombosis) and for epidermal growth factor (severe rash) were not seen in this clinical study.

#### Vatalanib

Vatalanib (formerly known as PTK787/ZK 222584) is an oral inhibitor of VEGFR-1, VEGFR-2, and VEGFR-3 TKIs and other related kinases such as PDGFR-β, c-KIT, and c-Fms [112]. *In vivo* studies of vatalanib in mice showed that this agent significantly inhibited growth in many types of tumors and had the potential to inhibit metastasis [117]. Pharmacokinetic results for doses up to 1,000 mg/day showed that vatalanib used once a day is rapidly absorbed, with a time of maximum concentration of 1.5 hours and a terminal half-life of about 3 to 6 hours [118].

In a view of vatalanib’s short half-life, subsequent studies explored twice-a-day administration. A phase I study in patients with advanced solid tumors using doses of oral vatalanib at 150 to 1,000 mg twice a day established that the maximum tolerated oral dose was 750 mg twice a day, whereas the biologically activity dose was more than 1,000 mg twice a day [119]. The DLT of reversible grade 3 lightheadedness was observed, along with dose-related grade 3 fatigue and vomiting. In phase I studies, promising antitumor activity was observed in patients with metastatic colorectal cancer.

### Farnesyltransferase inhibitors

Our understanding of the molecular biology of Ras and its downstream pathways has grown considerably during the past decades. Ras proteins play a pivotal role in the transduction of cell growth-stimulating signals, and the mutation of the *ras* gene leads to constant activation of the protein, resulting in uncontrolled cell proliferation [120]. Point mutations in the *ras* proto-oncogene therefore result in permanently active Ras and are oncogenic. Although fewer than 5% of breast cancers have *ras* mutations, hyperactivation of the Ras protein in breast cancer has been described [121]. Rho proteins, downstream effectors of Ras, control cytoskeleton reorganization and gene expression. Overexpression of Rho was associated with locoregional and distant metastasis of breast cancer [122] and with inflammatory breast cancer [123].

Several compounds in preclinical and clinical trials have targeted various stages of the Ras signaling cascade, including inhibition of Ras expression via antisense oligo-deoxynucleotides, interference via farnesyltransferase inhibitors, and inhibition of Ras downstream effectors via MEK, PI3K inhibitors, and others. The most advanced farnesyltransferase inhibitors currently in clinical development are tipifarnib and lorafarnib (SCH66336).

#### Tipifarnib

Tipifarnib (formerly known as R115777) is an imidazole-containing heterocyclic compound that inhibits the growth of several wild-type and *ras*-mutated tumor cell lines and inhibits the growth of tumor xenografts in a dose-dependent manner, including wild-type *ras* MCF-7 breast cancer cells [124]. In phase I trials, tipifarnib has been administered at doses up to 1,300 mg twice daily for 5 days every 2 weeks without significant toxicity [125]. In a phase II study of tipifarnib in patients with ER-positive MBC that progressed during second-line hormone therapy, 25% of patients achieved CB [126].

Tipifarnib was combined with dose-dense doxorubicin and cyclophosphamide as neoadjuvant therapy for patients with locally advanced breast cancer, and seven out of 21 patients had a pathologic complete response [127]. These results are very encouraging because the pathologic complete responses occurred in ER-positive patients. In the recent publication of a phase II study in front-line therapy for MBC, tipifarnib combined with fulvestrant resulted in a CB rate of 51.6% [128].

### Mammalian target of rapamycin inhibitors

The PI3K signaling pathway is crucial to many key cellular functions, including growth, proliferation, survival, angiogenesis, and motility [129]. Aberrant activation of the pathway contributes to tumorigenesis, tumor metastases, and resistance to standard cancer therapy. In contrast to p53 and other tumor-suppressor pathways, the PI3K pathway is activated in cancer, making this an optimal target for therapy. PI3Ks are classified into three classes on the basis of their primary structure and substrate specificity: everolimus, sirolimus, and temsirolimus [130].

#### Everolimus

Everolimus (known as RAD-001) has greater polarity than sirolimus and was developed in an attempt to improve the pharmacokinetic characteristics of sirolimus, particularly to increase its oral bioavailability. Several studies showed that the most common toxicity observed with everolimus were diarrhea, asthenia, hyperglycemia, and anemia. A phase II, double-blind, randomized, placebo-controlled trial evaluated the value of adding everolimus to letrozole as primary systemic therapy [131]. The study showed that the combination of everolimus plus letrozole was associated with a higher ORR (68.1% vs. 59.1%), which was confirmed by ultrasound (58% vs. 47%).

#### Temsirolimus

Temsirolimus (known as CCI-779) is a water-soluble ester of sirolimus. In preclinical studies, temsirolimus has demonstrated antitumor activity in breast cancer models [132]. In a phase I, dose/schedule-finding study in patients with advanced malignancies, 24 patients were treated with temsirolimus with doses ranging from 7.5 to 220 mg/m^2^ as a weekly intravenous infusion [133]. A DLT, thrombocytopenia, occurred in two patients at 34 or 45 mg/m^2^ and at 220 mg/m^2^. The most common related adverse events were dermatologic toxicity, and mucositis was seen in 71% of the patients. Other DLTs consisted of manic-depressive syndrome, stomatitis, and asthenia. All toxicities were reversible after treatment discontinuation. Two patients with renal cell carcinoma and breast cancer achieved PR.

In an international phase II study, patients previously treated for locally advanced breast cancer or MBC were randomized 1:1 to receive intravenous temsirolimus weekly at a dose of 75 or 250 mg [134]. A total of 109 patients participated in the study. For at least 24 weeks (per Response Evaluation Criteria in Solid Tumors), CB was observed in 13.8% of patients; 10 patients had PR, and the ORR was 9.2%. The most common adverse effects were mucositis (70%), maculopapular rash (51%), and nausea (43%). Both doses showed antitumor activity, and 75 mg generally resulted in a tolerable safety profile.

### Poly (ADP-ribose) polymerase inhibitors

Poly (ADP-ribose) polymerase (PARP) 1 is a critical enzyme of cell proliferation and DNA repair. Multiple PARP-1 inhibitors have been tested preclinically as potentiators of chemotherapy and radiotherapy [135]. One function of PARP enzymes, particularly PARP-1 and PARP-2, is in the repair of single-stranded DNA breaks [136].

Given that *BRCA1* -related breast cancers generally have the same phenotypic expression profiles as BRCA-negative basal breast cancers, it has been hypothesized that sporadic TRN breast cancers may have a DNA repair deficit similar to that in BRCA-mutant cases. A randomized phase II study of BSI-201 in combination with gemcitabine plus carboplatin demonstrated that the combination prolonged both PFS and OS in TRN MBC [137]. In total, 123 patients with TRN MBC were randomized to receive gemcitabine/carboplatin with or without BSI-201. Gene expression profiling performed primarily on breast cancer samples from 50 patients showed that PARP-1 expression was significantly upregulated (*P* <0.0001). In the preliminary analysis, the CB rate was significantly better with BSI-201 plus gemcitabine/carboplatin than with gemcitabine/carboplatin alone (62% vs. 21%, respectively; *P =* 0.0002), as were the ORR (48% vs. 16%, respectively; *P =* 0.0001) and the median OS (9.2 vs. 5.7 months; *P =* 0.0005). There were no significant toxicity differences between treatment arms. The promising efficacy and low toxicity results have prompted the initiation of a phase III study.

Olaparib (AZD2281) is a novel PARP inhibitor with significant activity in patients with mutation of *BRCA1/2* breast cancer, ovarian cancer, or prostate cancer [131]. Preliminary results from a multicenter, open-label, phase II trial of olaparib in heavily pretreated patients with *BRCA1/BRCA2*-mutated advanced breast cancer were presented at the American Society of Clinical Oncology in 2009 [138]. This single-arm study included two sequential cohorts of patients: 27 patients who received 400 mg olaparib twice daily and 27 patients who received 100 mg twice daily. The ORR was 41% or 22% for those given 400 mg or 200 mg, respectively. The cohort of patients treated with higher doses also showed improvement in other clinical end points: the median PFS time was longer with 400 mg versus 200 mg at 5.7 months versus 3.8 months, respectively, and most of the patients receiving 400 mg twice daily experienced tumor shrinkage. There were no differences in toxicity between the two arms.

Several phase II studies using other PARP inhibitors (ABT-888, AGO14699, and MK4827) are also being investigated in early-stage trials.

## Conclusion

The most recent major contribution to the treatment of breast cancer has not been a technical or pharmacological revolution, but rather a transformation in the way we think about the disease and the treatment. Biotechnology advances that facilitated the development of new therapeutic drugs were accompanied by an explosion of interest in the large-scale study of gene expression patterns. The development of new drugs in oncology, however, faces multiple challenges in the new molecular era. The continuous application of the old paradigm of traditional schemas of response to new targeted therapies may be inaccurate since neither tumor response nor toxicity is a useful surrogate for dose selection or efficacy. We need a better understanding of the molecular biology of signaling pathways and we need to discover new biomarkers in order to select optimal doses in phase II clinical studies. In addition, the selection of patients for targeted therapy remains a challenge because we presently lack reliable biomarkers to predict activity for most of the targeted agents.

## Abbreviations

CB: clinical benefit; CI: confidence interval; DLT: dose-limiting toxicity; EGFR: epidermal growth factor receptor; ER: estrogen receptor; HER2: human epidermal growth factor receptor 2; HR: hazard ratio; IC_50_: half-maximal inhibitory concentration; IFN: interferon; MBC: metastatic breast cancer; ORR: overall response rate; OS: overall survival; PARP: poly(ADP-ribose) polymerase; PDGFR: platelet-derived growth factor receptor; PFS: progression-free survival; PI3K: phosphatidylinositol-3-kinase; PR: partial response; SABCS: San Antonio Breast Cancer Symposium; SD: stable disease; TKI: tyrosine kinase inhibitor; TRN: triple receptor negative; VEGF: vascular endothelial growth factor; VEGFR: vascular endothelial growth factor receptor.
